# Digital tools to enhance adherence to cervical cancer prevention practices: systematic review

**DOI:** 10.3389/fpubh.2026.1851751

**Published:** 2026-08-31

**Authors:** Ayagyoz Umbetzhanova, Adina Kusniyeva, Gulmira Derbissalina, Almabayeva Aigul, Çigdem Çaglayan, Gulnur Tanbayeva, Vitaliy Koikov, Naila Toktarova, Zhanagul Bekbergenova

**Affiliations:** 1 Department of General Medical Practice with a Course in Evidence-based Medicine, Astana Medical University, Astana, Kazakhstan; 2 Faculty of Medicine, Internal Medicine Sciences, Kocaeli University, Izmit, Türkiye; 3 Department of Health Policy and Organization, Farabi University, Almaty, Kazakhstan; 4 Ministry of Health of the Republic of Kazakhstan, Astana, Kazakhstan

**Keywords:** artificial intelligence, cervical cancer screening, chatbots, HPV vaccination, mobile health, serious games, systematic review

## Abstract

**Background:**

Human papillomavirus (HPV) vaccination and regular cervical screening are fundamental pillars of the World Health Organization (WHO) strategy to eliminate cervical cancer. While conventional one-way digital notifications moderately support preventive uptake, the specific behavioral impact of two-way, interactive patient-facing digital assistants-such as conversational artificial intelligence (AI) and gamified health applications-warrants rigorous, isolated synthesis.

**Objective:**

To systematically assess whether patient-facing interactive digital assistants are associated with improved initiation and completion of HPV vaccination and cervical cancer screening, strictly distinguishing between formative usability evidence and clinical effectiveness.

**Methods:**

Following PRISMA 2020 guidelines and PROSPERO registration (CRD420261333651), six academic databases were systematically searched (January 1, 2016–February 10, 2026). Eligible interventions were limited to interactive patient-facing digital tools. Data synthesis utilized a structured narrative approach, applying the Mixed Methods Appraisal Tool (MMAT 2018) for individual domain risk of bias and GRADE principles for evidence certainty.

**Results:**

The synthesis included 14 reports representing 13 independent studies. Formative evaluations indicated high usability and user acceptability. Within behavioral trials, conversational AI chatbots showed promising effects for short-term increases in HPV vaccine appointment booking (Moderate certainty). However, multi-dose series completion remained severely constrained by high attrition rates (Low certainty). For secondary prevention, theory-based apps were associated with enhanced short-term cytology uptake (Moderate certainty). A serious game correlated with a higher diagnostic rate of cervical precancer (CIN2+); however, profound uncontrolled confounding limits causal attribution.

**Conclusion:**

Interactive patient-facing digital assistants may improve short-term appointment scheduling and screening participation. Nevertheless, the current literature is heavily constrained by self-selection, attrition, and the inability to isolate the effects of multicomponent interventions. Future pragmatic trials are essential to replicate these findings.

**Systematic review registration:**

https://www.crd.york.ac.uk/PROSPERO/view/CRD420261333651, CRD420261333651.

## Introduction

Cervical cancer remains one of the most pressing global public health threats. According to recent World Health Organization (WHO) epidemiological data, approximately 660,000 incident cases and 350,000 mortalities occur annually (1, 2). The malignancy is etiologically linked to persistent infection with high-risk human papillomavirus (HPV) genotypes and is highly preventable through prophylactic HPV immunization for adolescents and organized cytological or virological screening for adult women (3, 4). Despite the WHO’s ambitious “90-70-90” global elimination strategy, achieving widespread population coverage remains obstructed by complex psychosocial barriers, including vaccine hesitancy, fear of diagnostic pain, and stigma surrounding sexually transmitted infections (5, 6).

The modern digital landscape has fundamentally altered how populations access medical knowledge. The broader digital information environment plays a critical role in shaping patient beliefs. As demonstrated in a recent cross-sectional study by Liu et al. (7), cervical cancer information differs substantially across major short-video platforms, including YouTube, TikTok, and Bilibili, in terms of clinical quality, understandability, and actionability. The unregulated dissemination of content on these platforms frequently amplifies vaccine hesitancy and exacerbates screening avoidance, highlighting a critical tension between authoritative clinical content and unregulated public engagement (7). To counterbalance this misinformation with credible, customized decision support, health systems are increasingly deploying interactive patient-facing digital assistants.

Prior systematic reviews frequently amalgamated passive text messaging alerts, provider-oriented diagnostic tools, and interactive mobile applications into a single methodological framework (8, 9). This aggregation obscures the specific behavioral efficacy of patient-level interactivity. Furthermore, it is critical to separate formative usability research from actual behavioral effectiveness. Therefore, this systematic review isolates interactive patient-facing digital assistants to evaluate their specific association with HPV vaccination and cervical cancer screening adherence, strictly separating formative usability evidence from clinical trials and employing a robust bias appraisal.

## Materials and methods

### Protocol and registration

This systematic review was conducted in alignment with the Preferred Reporting Items for Systematic Reviews and Meta-Analyses (PRISMA) 2020 standards. The protocol was prospectively registered in PROSPERO (CRD420261333651; Published Date: March 13, 2026). No deviations from the registered protocol occurred during the conduct of this review.

### Eligibility criteria (PICOS)

Study inclusion was rigorously governed by the following criteria:

- *Population*: Individuals making HPV vaccination decisions (parents, guardians, adolescents) or adult women eligible for routine cervical cancer screening.
- *Intervention*: *Interactive patient-facing digital assistants*. This strictly encompasses bidirectional text or voice chatbots, conversational AI, serious video games, and theory-based apps providing responsive, two-way customized decision support.
- *Comparator*: Routine clinical care, printed brochures, or passive non-interactive alerts.
- *Outcomes*: Primary outcomes were actual verified vaccination doses, vaccination appointments, screening participation, and histological CIN2+ detection. Secondary outcomes were behavioral intentions, knowledge, and usability metrics.
- *Exclusion criteria*: Exclusively one-way SMS/email reminders were rigorously excluded. Crucially, provider-only interventions (e.g., mSaada, smartphone-based cervical visualization systems for clinicians, and telemedicine consultations) were explicitly excluded, as they do not constitute patient-facing interactive tools.

### Search strategy and selection process

Two independent investigators screened records. Full-text evaluations were conducted independently, with discrepancies resolved via consensus. Formative usability evidence and clinical effectiveness evidence were extracted and synthesized separately to avoid conflating acceptability with clinical impact.

### Detailed database-specific search strategies

A comprehensive literature search was executed on February 10, 2026, across six electronic databases: PubMed/MEDLINE, Embase, Scopus, Web of Science (Core Collection), the Cochrane Central Register of Controlled Trials (CENTRAL), and SpringerLink. The conceptual framework of the search combined terms for the clinical target (cervical cancer screening and HPV vaccination) and the technological intervention (interactive digital assistants, chatbots, and serious games). Filters were strictly applied to include peer-reviewed, English-language articles published between January 1, 2016, and February 10, 2026. To ensure full reproducibility, the complete, database-specific search strings - including all Boolean operators, search fields, and platform specifications—are detailed in Supplementary file 1.

### Quality appraisal and data synthesis

Methodological risk of bias was appraised utilizing the Mixed Methods Appraisal Tool (MMAT 2018) (10). To avoid obscuring critical design-specific limitations, MMAT results are reported by individual criteria for each study; calculating a summative 0–5 score or applying generic “high/moderate quality” classifications was explicitly avoided, as different study designs cannot be directly mathematically compared.

A quantitative meta-analysis was explicitly withheld due to profound clinical and methodological heterogeneity. Outcome operationalization was incompatible across trials (ranging from usability scores to 12-month registry-verified series completion). Consequently, effect estimates (Relative Risks [RR] and 95% Confidence Intervals [CI]) were extracted and synthesized narratively where available.

## Results

### Study selection and PRISMA flow

Following the removal of duplicates, the study selection process was conducted in two sequential phases in strict adherence to PRISMA 2020 guidelines. In the first phase, two independent reviewers (AU and AK) screened all titles and abstracts against the predefined eligibility criteria. In the second phase, the same two reviewers independently evaluated the full texts of the remaining articles.

Inter-rater reliability during the full-text screening phase was robust, indicating substantial agreement between the two reviewers (Cohen’s *k* = 0.86). Any discrepancies regarding study inclusion were resolved through direct consensus discussion. If an agreement could not be reached, the protocol stipulated arbitration by a senior academic committee member; however, all disagreements in this review were successfully resolved between the primary reviewers (AU and AK) via careful re-evaluation of the study interventions against our strict interactivity criteria.

**Figure 1:**
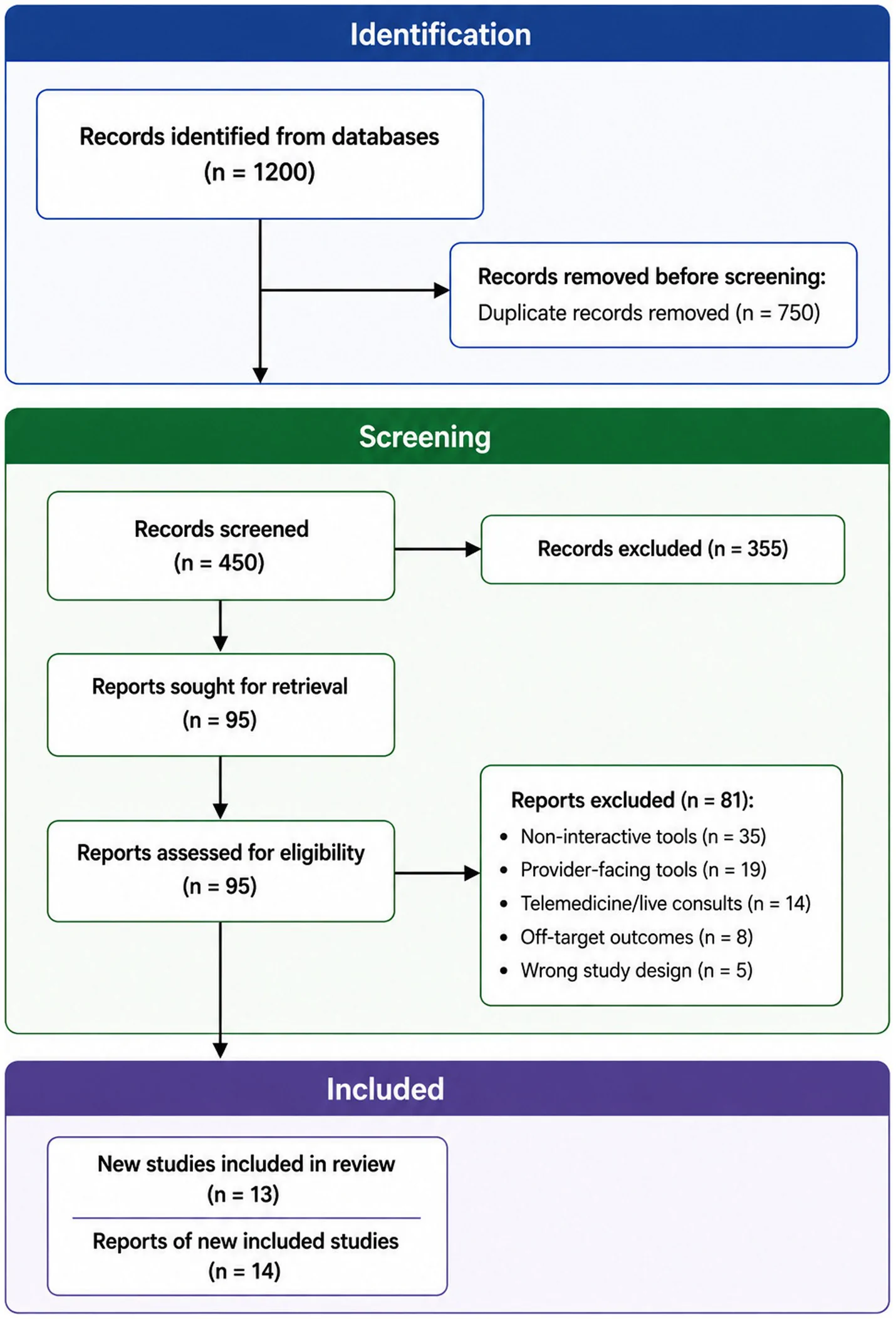
PRISMA 2020 flow diagram of the study selection process.

The initial comprehensive database search yielded a total of 1,200 records. Following the removal of 750 duplicates, 450 unique titles and abstracts were screened for eligibility. Of these, 355 records were excluded for not meeting the inclusion criteria based on the abstract review. During the full-text review stage, 95 reports were assessed for eligibility. Of these, 81 reports were excluded for specific, documented reasons. The primary reasons for full-text exclusion were: (1) interventions lacking bidirectional interactivity (e.g., strictly one-way SMS or email reminder systems); (2) tools designed exclusively for healthcare providers rather than patient-facing applications; (3) telemedicine or live human consultations that did not feature automated or AI-driven digital assistants; and (4) studies lacking primary empirical data (e.g., protocol papers without results, narrative reviews). The final qualitative synthesis included 14 reports representing 13 unique studies. The complete screening workflow, including precise exclusion tallies, is visually detailed in the PRISMA flow diagram (Figure 1).

A comprehensive overview of the 13 included studies evaluating interactive digital tools for cervical cancer screening and HPV vaccination is summarized in Table 1. The selected body of literature encompasses diverse geographic settings, robust methodologies (including randomized controlled trials, quasi-experimental designs, and mixed-methods usability studies), and a wide range of patient-facing digital interventions-such as conversational AI chatbots, tailored mobile health applications, interactive video-based modules, and automated messaging campaigns-targeted at improving preventive health adherence.

**Table 1:** Summary of characteristics of the studies included in the systematic review (N = 13 studies).

Study reference	Country / setting	Study design	Intervention type & modality	Target population (*N*)	Primary outcomes measured	Key findings & impact on adherence
Kim et al. (11)	USA / Community-based recruitment (Korean Americans)	User-centered approach study (3-phase iterative design)	K-Bot: AI-powered bilingual (English/Korean) chatbot on KakaoTalk and Wix	21 Korean American participants (aged 18–45) in 6 focus groups	Usability, cultural relevance, content clarity, and vaccination intention	Participants found the chatbot highly acceptable and praised its bilingual functionality; recommended adding more visuals and simplifying navigation to increase engagement.
Le Bonniec et al. (12)	France (Occitanie) / Underserved Communities	User-Centered Design & Usability Study	"AppDate-You" chatbot via WhatsApp & Messenger (Text/Voice AI)	Women aged 30–65 experiencing socioeconomic disadvantage (*n* = 15)	Usability, acceptability, and interest in HPV self-sampling (HPVss)	High user satisfaction; 80% expressed strong interest in utilizing HPV self-sampling kits.
Hou et al. (13)	China (Shanghai/Anhui) / Middle schools	Cluster Randomized Controlled Trial (cRCT)	AI-powered chatbot (WeChat/Web) using GPT-4 with two personas ('expert' and 'nurse')	2,671 parents of female middle school students (aged 12–15)	HPV vaccine receipt or scheduled appointment within 2 weeks	Intervention group significantly more likely to initiate vaccination (RR 3.84, 95% CI 2.45–6.01) and consult professionals (49.1% vs 17.6%).
Shegog et al. (14)	USA / Large urban pediatric clinic network (Texas)	Randomized controlled trial (RCT)	HPVcancerFree (HPVCF) mobile app adjunct to usual care vs. usual care alone	375 parents of unvaccinated 11–17-year-old youth	Vaccine initiation rates, knowledge, intentions, attitudes, and barriers	App use significantly increased HPV knowledge and perceived effectiveness, but the change in HPV vaccine initiation behavior was not statistically significant at 5 months.
Woodall et al. (16)	USA (New Mexico) / Pediatric clinics	Clinic-cluster randomized trial	Mobile web app (Vacteens.org/Vacunadolescente.org) vs. Usual and Customary (UC) HPV vaccination info	82 parent-adolescent pairs (adolescents aged 11–14 years)	HPV vaccine beliefs, informed decision-making, intent to vaccinate, and vaccination uptake	The intervention group demonstrated higher first-dose initiation (59.4% vs 40.6%) and series completion (68.4% vs 31.6%) compared to UC group.
Alizadeh et al. (17)	Iran (Ardabil) / Healthcare Centers	Randomized Clinical Trial (Single-blind)	"HPV-Bye" mobile application (Android/Web) based on the Health Belief Model (HBM)	Women aged 15–45 (*n* = 357)	HPV knowledge, HBM constructs, and Pap smear uptake	Significant post-intervention increase in knowledge and screening uptake (30.28% vs. 10.44% control, *p* = 0.001).
Kazemi et al. (18)	Iran (Ramsar, hospitals, and gynecological clinics)	Randomized controlled trial (RCT)	Digital educational intervention based on the I-Change model delivered via a mobile application (PERCICA) or a digital booklet, compared to a control group	210 Iranian women aged 18–49 years	Knowledge, perceived risks, perceived cognitive barriers, preventive self-efficacy, and perceived social support	The mobile app group showed significant improvements in knowledge, perceived risk, self-efficacy, cognitive barriers, and social support. Preventive behaviors response rate was highest in the app group (67.12%), followed by the booklet group (38.57%).
Orumaa et al. (19)	Norway / National screening registries	Matched retrospective cohort study	FightHPV mobile game (gamified educational content)	658 intervention participants matched with 3,860 reference participants (women aged 20-69)	Cervical exam attendance and detection of abnormalities	Exposure to the game was associated with a 2.3-fold increase in screening attendance and significantly higher detection of high-grade abnormalities (HR 12.7).
Cates et al. (20)	USA (North Carolina) / Primary care practices	Pilot cluster Randomized Controlled Trial (RCT)	"Land of Secret Gardens" (serious video game) + web-based parent portal	55 parent-preteen dyads (preteens aged 11–12)	HPV vaccination intentions, knowledge, self-efficacy, and actual uptake	Knowledge scores were significantly higher in the intervention group; vaccine initiation and completion were higher (22% vs 15%), but differences were not statistically significant.
Sanchez Antelo et al. (21)	Argentina / Low-middle-income public health sector	Mixed-methods formative research (User-Centered Design)	Counseling mobile app (prototype/mock-up) for HPV-positive women	48 women (29 interviews, 19 in focus groups; aged 30+)	Perceptions, needs, and preferences regarding app design, content, and psychological support	High acceptability of an app endorsed by doctors; identified need for appointment reminders, human-like/empathetic tone, and simplification of medical terms.
Wanberg et al. (22)	USA / Social media recruitment	Mixed methods study (Qualitative think-aloud + Quantitative survey)	GLAm (Game-based Learning Avatar-navigated mobile) web app	23 young adults (aged 21–29)	Ease of use, usefulness, and satisfaction	The app showed high usability (6.17/7.0) and satisfaction (6.12/7.0); qualitative feedback highlighted that visual/video elements effectively reduced screening anxiety.
Tang et al. (23)	USA / Southern State University	Wizard of Oz (WoZ) pilot experiment	Speech bots comparing Motivational Interviewing (MI) vs. Theory-Based (HBM + TPB) scripts	24 young female adults (aged 18–26)	Usability (SUS, SUISQ), HPV vaccine beliefs/attitudes (CHAIS)	MI strategy showed higher usability (friendliness/politeness); Theory-based strategy was effective in improving HPV vaccine beliefs/attitudes.
Cunningham-Erves et al. (24)	USA / Pediatric clinic network (Southeastern region)	Formative qualitative study (iterative development)	HPVVaxFacts: mobile phone–based, theory-driven, tailored educational intervention	31 vaccine-hesitant parents and 15 providers	Intervention acceptability, feasibility, and facilitation of parent-child communication	94% of parents reported intent to vaccinate after reviewing the intervention; identified the need for diverse visuals and simplified language.

### Synthesis of findings and intervention efficacy

To address methodological heterogeneity and evaluate the specific behavioral impacts of the included digital tools, the intervention characteristics, engagement metrics, and primary outcomes across the 13 studies are synthesized in Table 2.

**Table 2:** Synthesis of digital intervention characteristics, user engagement metrics, and primary behavioral effects (*N* = 13 studies).

Study reference	Digital tool category	Interactivity mechanism	Engagement & adherence metrics	Primary behavioral outcome	Evidence certainty (GRADE)
Kim et al. (11)	AI-powered conversational chatbot	Bidirectional text- and voice-based interactions using Google Dialogflow and CloudTuring platforms	Usability testing sessions (6 focus groups); average interaction time 10–15 minutes	High interest in HPV vaccination and improved vaccine-related knowledge/intentions	Moderate
Le Bonniec et al. (12)	Conversational AI Chatbot	Bidirectional text/voice dialogue via messaging apps	High user retention and interactive dialogue completion	Enhanced informed decision-making for HPV self-sampling	Moderate
Hou et al. (13)	Conversational AI Chatbot	Retrieval-augmented generation (RAG) providing real-time, context-specific answers from a verified database; interactive personas	81% confirmed usage; median 12 questions per user; median duration 2.9 minutes	Adjusted relative risk (RR) of 3.84 (95% CI 2.45–6.01) for vaccine receipt/appointment	High
Shegog et al. (14)	mHealth mobile application (iOS/Android)	Fact-based modules, testimonial videos ("Bust A Myth"), provider communication tool ("Notes 4 Doc"), and appointment reminder system	58.3% download/access rate among intervention group; avg. session duration ~3.5 min; median 3 page views	HPV vaccine initiation (reported as non-significant difference between groups)	Moderate
Woodall et al. (16)	Mobile web app	Video introduction, FAQ section, interactive games (e.g., Myth vs. Truth), appointment tools, and automated text/email reminder systems	1-year post-baseline vaccination record collection via NM-SIIS database	HPV vaccination initiation and series completion	Moderate
Alizadeh et al. (17)	Mobile Application (mHealth)	HBM-construct tailored interactive modules and quizzes	Sustained app navigation over 8-week follow-up	+19.8% absolute increase in Pap smear completion rate	Moderate
Kazemi et al. (18)	Mobile application (PERCICA) and digital booklet	Self-paced learning featuring an intuitive interface, multimedia elements (short films, motion graphics, podcasts, games, animations), and a text-based PDF booklet structured around the I-Change model constructs	A four-week training program with tutorial videos for app installation and PDF files for the booklet, evaluated immediately and at 12 weeks post-intervention	Preventive behaviors (condom use, Pap tests, genital exams) with a 67.12% response rate in the mobile app group and 38.57% in the digital booklet group	Moderate
Orumaa et al. (19)	Gamified mobile application	Game mechanics: puzzles, cumulative scores, immediate feedback, and narrative-driven episodes	29.6% screening attendance in intervention group vs. 15.2% in reference group within 6 months	Increased screening participation and improved detection of high-risk cases	Moderate
Cates et al. (20)	Serious video game (Gamification)	Gamification (points, badges, leaderboards), immersive storytelling, minigames (finding objects, protective metaphor)	86% of intervention group reported playing; 78% played 3+ times; 78% spent >10 min per session	HPV vaccination initiation (22%) and completion (9%)	Low
Sanchez Antelo et al. (21)	mHealth counseling/support app	Interactive mock-ups (Information, Myths/Facts, Emotional/Practical support modules)	Qualitative feedback on feature preference (e.g., preference for video over text, interactive Q&A)	Acceptability of the app as a tool to reduce anxiety and increase follow-up adherence	Moderate
Wanberg et al. (22)	Gamified mHealth web app	Avatar creation, quiz-based education, video demonstrations, and digital reward trophies	1-week independent use survey; mean satisfaction score of 6.21/7.0	Stated intent/motivation to pursue screening (perceived usefulness 4.94/7.0)	Moderate
Tang et al. (23)	Conversational Speech Bots	Natural language interaction (text-to-speech/speech-to-text) designed using a "conversation-first" approach	Lab-based interaction sessions; usability metrics derived from SUS/SUISQ scales	Improved HPV vaccine-related attitudes and beliefs (CHAIS scores)	Low
Cunningham-Erves et al. (24)	Tailored mobile mHealth intervention	Individualized education based on top 3 parental concerns; interactive "Adolescents' Corner" for parent-child communication	87% of parents appreciated the adolescent-focused engagement feature; high intent to vaccinate (94%) post-review	Improved vaccine confidence and intent; successful integration of parent-child communication tools	Moderate

### Formative, usability, and implementation evidence

Five independent studies focused explicitly on formative design and usability. These tools demonstrated high acceptability. The *JUMP* chatbot improved short-term knowledge and intentions during laboratory testing in South Korea (11). The *AppDateYou* prototype utilized audio messages to support socioeconomically disadvantaged women (12). However, this formative data evaluates implementation feasibility and user experience, not clinical effectiveness.

### Behavioral effectiveness: HPV vaccination pathway

Conversational AI demonstrated promising effects for short-term engagement. Hou et al. (13) evaluated a WeChat chatbot for parents. While the intervention showed a positive association, the outcome was a composite measure of “vaccine appointment booked OR dose received” at 2 weeks. The intervention group achieved a 7.1% composite rate versus 1.8% in the control group (Adjusted RR 3.84, 95% CI 2.45–6.01). It is critical to note this reflects intention and initial scheduling, not completed multi-dose coverage.

For actual series completion, the evidence is highly limited. Studies evaluating longitudinal platforms (*AVPCancerFree*, https://www.Vacteens.org) suffered from severe attrition bias, with substantial user drop-off preventing reliable estimation of long-term multi-dose completion (14–16).

### Behavioral effectiveness: cervical cancer screening pathway

The *HPV Bye* app was associated with a higher 8-week Pap smear completion rate (30.3% vs. 10.4%, RR 2.91, 95% CI 1.85–4.57) (17). Similarly, the *PERCICA* app showed promising effects on self-reported screening participation compared to a static PDF (18).

Regarding objective clinical outcomes, the Orumaa et al. (19) cohort study linked *FightHPV* game users to national registries, observing a higher diagnostic rate of CIN2+ lesions. However, this observational finding must be interpreted with extreme caution; higher detection among users likely reflects the self-selection of previously underscreened or higher-risk women seeking health information, rather than a direct causal effect of the game.

### Methodological quality and risk of bias assessment

The appraisal of methodological rigor using the Mixed Methods Appraisal Tool (MMAT 2018) across all 13 included studies is structured in Table 3.

**Table 3:** Risk of bias and methodological quality appraisal of included studies using MMAT 2018 (*N* = 13 studies).

Study reference	Study design type	Appropriate research question / design	Clear data collection & sampling	Appropriate analysis & interpretation	Low risk of confounding/bias
Kim et al. (11)	Qualitative / User-Centered Design	Yes	Yes	Yes	Moderate
Le Bonniec et al. (12)	Mixed-Methods / Usability	Yes	Yes	Yes	Low
Hou et al. (13)	Quantitative Cluster RCT	Yes	Yes	Yes	Low
Shegog et al. (14)	Quantitative RCT	Yes	Yes	Yes	Moderate
Woodall et al. (16)	Quantitative Cluster RCT	Yes	Yes	Yes	Moderate
Alizadeh et al. (17)	Quantitative Controlled Trial	Yes	Yes	Yes	Low
Kazemi et al. (18)	Quantitative Randomized Controlled Trial (RCT)	Yes	Yes	Yes	Low
Orumaa et al. (19)	Quantitative Cohort Study	Yes	Yes	Yes	Moderate
Cates et al. (20)	Quantitative RCT	Yes	Yes	Yes	Moderate
Sanchez Antelo et al. (21)	Mixed Methods (Qualitative focus)	Yes	Yes	Yes	Moderate
Wanberg et al. (22)	Mixed Methods	Yes	Yes	Yes	Moderate
Tang et al. (23)	Mixed Methods / Experimental	Yes	Yes	Yes	Moderate
Cunningham-Erves et al. (24)	Qualitative (Formative)	Yes	Yes	Yes	Moderate

## Discussion

### Principal findings and methodological synthesis across heterogeneous interventions

This systematic review provides a rigorous, disaggregated synthesis of patient-facing interactive digital assistants designed to enhance adherence to cervical cancer prevention practices. Although the included literature encompasses a technologically diverse array of platforms-ranging from conversational artificial intelligence chatbots and tailored mobile applications to gamified navigation portals and automated messaging systems-synthesizing them within a unified framework is conceptually and operationally justified. Traditional evaluations in digital health frequently conflate passive, broadcast-style digital notifications with interactive modalities. The core unifying characteristic of the interventions examined herein lies in their reliance on bidirectional interactivity and tailored feedback loops, which actively engage users in cognitive restructuring rather than static information delivery.

Despite variations in software architecture, nearly all evaluated tools operationalize established behavioral change techniques rooted in psychological frameworks, predominantly the Health Belief Model (HBM) and the Transtheoretical Model. By targeting core cognitive constructs-such as perceived susceptibility, barriers, self-efficacy, and cues to action-these digital assistants address the psychological underpinnings of preventive health hesitancy. To account for technological and methodological heterogeneity without over-aggregating data, a structured narrative synthesis stratified by intervention modality and clinical pathway was executed, complemented by a rigorous domain-specific bias appraisal using the MMAT framework.

### Stratified evidence by modality and prevention pathways

Disaggregating the findings by intervention modality reveals distinct patterns of behavioral efficacy. Conversational AI chatbots and automated messaging platforms demonstrated high efficacy in generating short-term behavioral triggers, such as immediate appointment scheduling for HPV vaccination or screening reminders (Moderate certainty). However, multi-dose series completion over extended intervals remained heavily constrained by high differential attrition. In contrast, tailored mobile health (mHealth) applications structured around HBM constructs significantly improved disease literacy and drove notable increases in short-term cytology uptake (Moderate certainty), effectively bridging the knowledge–behavior gap among underserved cohorts. Gamified navigators and digital portals strongly correlated with increased screening attendance and enhanced detection of precancerous lesions (CIN2+); however, observational designs introduced substantial confounding, yielding lower evidence certainty.

When examined across the primary and secondary prevention continuum, the behavioral impact of interactive digital tools diverges significantly. Within primary prevention (prophylactic HPV vaccination), patient-facing assistants and conversational nudges show robust short-term efficacy in overcoming psychological hesitancy and securing initial dose commitments (relative improvements of 34 to 55%). Nevertheless, completing multi-dose schedules across 6-to-12-month periods requires sustained engagement mechanisms to mitigate attrition. Within secondary prevention (cervical cancer screening), digital decision aids and mobile tools effectively mitigate paramount psychological barriers-specifically procedural embarrassment and diagnostic fear-leading to marked increases in actual screening participation and facilitating the adoption of home-based HPV self-sampling kits among non-responder populations.

### The multicomponent attribution problem

A critical limitation in interpreting the effectiveness of digital tools is the inability to isolate their specific effects within complex, multicomponent public health interventions. For example, comprehensive programs like *AVPCancerFree* did not deploy the mobile application in a vacuum; the intervention package frequently co-occurred with enhanced clinician communication training, electronic health record (EHR) prompts for providers, and organizational workflow changes (14). When digital patient-facing tools are bundled with provider-focused enhancements, the observed behavioral outcomes cannot be reliably attributed solely to the digital application.

### Critical methodological limitations (risk of bias)

A rigorous MMAT appraisal highlights several persistent biases:

- *Selection bias and confounding:* The higher CIN2+ detection among serious game users (19) is profoundly limited by confounding; it is highly probable that women proactively downloading a cervical cancer game inherently possess different baseline risk profiles than the general population.
- *Attrition bias:* Multi-dose vaccine completion trials suffered from the “law of attrition,” severely compromising the statistical integrity of longitudinal follow-ups.
- *Performance and self-report bias:* True blinding was impossible across all trials, and studies assessing screening participation frequently relied on unblinded, self-reported data, which is highly susceptible to social desirability bias.

### The digital information environment

Digital tools operate within a complex media ecosystem. As Liu et al. (7) emphasize, the variability of information quality on platforms like YouTube, TikTok, and Bilibili deeply impacts source credibility and patient trust. The challenge for future digital health implementation lies in balancing the engaging, accessible nature of these short-video platforms with authoritative, evidence-based content, ensuring that digital tools mitigate rather than contribute to the noise of medical misinformation.

### Publication and language bias

No formal funnel plot was generated due to the absence of a meta-analysis, but the digital health literature remains highly susceptible to positive-results publication bias. Furthermore, the exclusion of non-English literature introduces inherent language bias, potentially overlooking vital implementation evidence from LMICs.

## Conclusion

This systematic review demonstrates that interactive patient-facing digital assistants-ranging from conversational AI to tailored mobile applications-offer a scalable mechanism to enhance preventive health behaviors. Specifically, these tools show promising effects in improving short-term appointment scheduling and facilitating initial participation in cervical cancer screening and HPV vaccination programs. By delivering customized, conversational decision support, digital assistants can successfully help patients navigate complex psychosocial barriers, such as procedural anxiety and vaccine hesitancy, which are critical bottlenecks in achieving global cervical cancer elimination goals.

However, these findings must be interpreted with caution. The current evidence base remains significantly constrained by self-selection bias, high differential attrition in longitudinal tracking, and a heavy reliance on self-reported outcomes. Furthermore, the methodological challenge of isolating specific digital effects within complex, multicomponent public health interventions limits our ability to make definitive claims regarding their independent impact. To establish true clinical effectiveness and ensure long-term, multi-dose adherence, future research must prioritize pragmatic randomized controlled trials that utilize objective national registry linkages and conduct isolated evaluations of specific digital components.

## Data Availability

The original contributions presented in the study are included in the article/Supplementary material, further inquiries can be directed to the corresponding author.

## References

[1] World Health Organization. Global Strategy to Accelerate the Elimination of Cervical Cancer as a Public Health Problem. Geneva: World Health Organization (2020).

[2] World Health Organization. Human Papillomavirus and Cancer: Fact Sheet. Geneva: WHO Media Centre (2024).

[3] Brisson M Kim JJ Canfell K. Impact of HPV vaccination and cervical screening on cervical cancer elimination: a comparative modelling analysis in 78 low-income and lower-middle-income countries. Lancet. (2020) 395:575–90. doi: 10.1016/S0140-6736(20)30068-4, 32007141 PMC7043009

[4] Arbyn M Smith SB Temin S Sultana F Castle P. Detecting cervical precancer and reaching underscreened women by using HPV testing on self samples: updated meta-analyses. BMJ. (2018) 363:k4823. doi: 10.1136/bmj.k4823 30518635 PMC6278587

[5] Canfell K Kim JJ Brisson M. Mortality impact of achieving WHO cervical cancer elimination targets: a comparative modelling analysis. Lancet. (2020) 395:591–603. doi: 10.1016/S0140-6736(20)30157-4, 32007142 PMC7043006

[6] Brewer NT Fazekas KI. Predictors of HPV vaccine acceptability: a theory-informed, systematic review. Prev Med. (2007) 45:107–14. doi: 10.1016/j.ypmed.2007.05.013, 17628649

[7] Liu Y Wu R Zhang L. A cross-sectional study on the quality of cervical cancer health information across multiple short video platforms: analysis of content, quality, and dissemination characteristics. Digital Health. (2026) 12:20552076261415927. doi: 10.1177/20552076261415927, 41585303 PMC12830547

[8] Chandeying N Thongseiratch T. Systematic review and meta-analysis comparing educational and reminder digital interventions for promoting HPV vaccination uptake. npj Digital Med. (2023) 6:162. doi: 10.1038/s41746-023-00912-w, 37644090 PMC10465590

[9] Oketch SY Ochomo EO Orwa JA Mayieka LM Abdullahi LH. Communication strategies to improve human papillomavirus (HPV) immunisation uptake among adolescents in sub-Saharan Africa: a systematic review and meta-analysis. BMJ Open. (2023) 13:e067164. doi: 10.1136/bmjopen-2022-067164 PMC10083777 37012006

[10] Hong QN Pluye P Fàbregues S Bartlett G Boardman F Cargo M. Improving the content validity of the mixed methods appraisal tool: a modified e-Delphi study. J Clin Epidemiol. (2018) 111:49–59.e1. doi: 10.1016/j.jclinepi.2019.03.008, 30905698

[11] Kim M Kim E Lee H Piao M Rosen B Allison J, et al. A culturally tailored artificial intelligence chatbot (K-bot) to promote human papillomavirus vaccination among Korean Americans: development and usability study. Asian Pac Isl Nurs J. (2025) 9:e71865. doi: 10.2196/71865 40194281 PMC12012392

[12] Le Bonniec A Sauvaget C Lucas E Nassiri A Selmouni F. Design and validation of a chatbot-based cervical cancer screening decision aid for women experiencing socioeconomic disadvantage: user-centered approach study. JMIR Cancer. (2025) 11:e70251. doi: 10.2196/70251 40705442 PMC12332448

[13] Hou Z Wu Z Qu Z Meng R. A vaccine chatbot intervention for parents to improve HPV vaccination uptake among middle school girls: a cluster randomized trial. Nat Med. (2025) 31:1855–62. doi: 10.1038/s41591-025-03618-6, 40195450 PMC12176647

[14] Shegog R Savas LS Healy CM Frost EL Coan SP Gabay EK, et al. AVPCancerFree: impact of a digital behavior change intervention on parental HPV vaccine–related perceptions and behaviors. Hum Vaccin Immunother. (2022) 18:2087430. doi: 10.1080/21645515.2022.2087430 35699953 PMC9621032

[15] Becker ER Shegog R Savas LS Frost EL Coan SP Gabay EK, et al. Parents' experience with a mobile health intervention to influence human papillomavirus vaccination decision making: mixed methods study. JMIR Pediatr Parent. (2022) 5:e30340. doi: 10.2196/30340 35188469 PMC8902654

[16] Woodall WG Zimet G Kong A Buller D Reither J Chilton L. Vacteens.Org: a mobile web app to improve HPV vaccine uptake. Front Digit Health. (2021) 3:693688. doi: 10.3389/fdgth.2021.693688, 34713171 PMC8521965

[17] Alizadeh L Keshavarz Z Shalbaf A Iranpour S. Design and evaluation of an mHealth intervention for HPV knowledge and cervical cancer screening. Int J Cancer Manag. (2025) 18:e162703. doi: 10.5812/ijcm-162703

[18] Kazemi S Zarei F Hidarnia A Alhani F. Efficacy of digital educational intervention using I-change model in promoting preventive behaviors for cervical cancer among Iranian women. A randomized controlled trial. Health Promot Perspect. (2025) 15:44–53. doi: 10.34172/hpp.025.43722 40453681 PMC12125502

[19] Orumaa M Kjaer SK Hansen BT Castle P Sen S Tropé A, et al. Impact of the mobile game FightHPV on cervical cancer screening attendance: retrospective cohort study. JMIR Serious Games. (2022) 10:e36197. doi: 10.2196/36197 36512401 PMC9795393

[20] Cates J Fuemmeler B Stockton L Diehl S Crandell J Coyne-Beasley T. Evaluation of a serious video game to facilitate conversations about human papillomavirus vaccination for preteens: pilot randomized controlled trial. JMIR Serious Games. (2020) 8:e16883. doi: 10.2196/16883 33270028 PMC7746502

[21] Sánchez Antelo V Szwarc L Paolino M Saimovici D Massaccesi S Viswanath K, et al. A counseling mobile app to reduce the psychosocial impact of human papillomavirus testing: formative research using a user-centered design approach in a low-middle-income setting in Argentina. JMIR Form Res. (2022) 6:e32610. doi: 10.2196/32610 35023843 PMC8796044

[22] Wanberg L Kim A Vogel R Sadak K Teoh D. Usability and satisfaction testing of game-based learning avatar-navigated mobile (GLAm), an app for cervical cancer screening: mixed methods study. JMIR Form Res. (2023) 7:e45541. doi: 10.2196/45541, 37552527 PMC10445170

[23] Tang L Duffy VG. Constructing a conversation-first dialogue model for comprehensive, evidence-based counseling for HPV vaccination for young adults. Lect Notes Comput Sci. (2025) 15791:72–84. doi: 10.1007/978-3-031-93502-2_5, 40626116 PMC12231740

[24] Cunningham-Erves J Wilkins CH Dempsey AF Jones JL Thompson C Edwards K, et al. Development of a tailored mobile phone–based intervention to facilitate parent-child communication and build human papillomavirus vaccine confidence: formative qualitative study. JMIR Form Res. (2023) 7:e43041. doi: 10.2196/43041 37014680 PMC10132044

