# Supplementary material for: Digital tools to enhance adherence to cervical cancer prevention practices: systematic review
Source: Front Public Health. 2026 Aug 31;14:1851751. doi: 10.3389/fpubh.2026.1851751 (PMC13572625; doi:10.3389/fpubh.2026.1851751)
Supplement: Supplementary file 1 [file Data_Sheet_1.docx]

Supplementary Material

# 1 Supplementary Data

**Supplementary File 1**.

**Methodological Overview** The comprehensive search strategy detailed below was executed on February 10, 2026. The conceptual framework of the search was built upon two primary domains: (1) the clinical target (Cervical Cancer Screening OR HPV Vaccination) AND (2) the technological intervention (Interactive Digital Assistants OR Chatbots OR Serious Games). To ensure the high certainty of evidence required for this systematic review, limits were strictly applied to exclude non-peer-reviewed gray literature, conference abstracts, and preprints.

- **Execution Date:** February 10, 2026
- **Date Range Filter Applied:** January 1, 2016 – February 10, 2026
- **Language Filter:** English only

**1. PubMed / MEDLINE** *Parameters applied: Medical Subject Headings (MeSH) combined with Title/Abstract (tiab) free-text terms.*

("Papillomavirus Vaccines"[MeSH] OR "Papillomavirus Infections/prevention and control"[MeSH] OR "Early Detection of Cancer"[MeSH] OR "Vaginal Smears"[MeSH] OR "HPV vaccin*"[tiab] OR "cervical cancer screening"[tiab] OR "pap smear"[tiab] OR "human papillomavirus"[tiab]) AND ("Artificial Intelligence"[MeSH] OR "User-Computer Interface"[MeSH] OR "Mobile Applications"[MeSH] OR "Video Games"[MeSH] OR "chatbot*"[tiab] OR "conversational agent*"[tiab] OR "conversational AI"[tiab] OR "serious game*"[tiab] OR "interactive app*"[tiab] OR "digital assistant*"[tiab] OR "gamif*"[tiab])

**2. Embase** *Parameters applied: Emtree terms (exp) combined with Title/Abstract/Keyword (ti,ab,kw) search fields.*

('papillomavirus vaccine'/exp OR 'cervical cancer screening'/exp OR 'papanicolaou test'/exp OR 'hpv vaccin*':ti,ab,kw OR 'cervical screening':ti,ab,kw) AND ('artificial intelligence'/exp OR 'human computer interaction'/exp OR 'mobile application'/exp OR 'chatbot*':ti,ab,kw OR 'conversational agent*':ti,ab,kw OR 'serious game*':ti,ab,kw OR 'interactive digital':ti,ab,kw OR 'virtual assistant*':ti,ab,kw) AND [english]/lim AND [2016-2026]/py

**3. Scopus** *Parameters applied: Title, Abstract, and Keyword (TITLE-ABS-KEY) overarching search fields.*

TITLE-ABS-KEY ( ( "HPV vaccin*" OR "cervical cancer screening" OR "pap smear" OR "pap test" ) AND ( "chatbot*" OR "conversational agent*" OR "artificial intelligence" OR "serious game*" OR "interactive mobile app*" OR "digital assistant*" OR "gamif*" ) ) AND PUBYEAR > 2015 AND LIMIT-TO ( LANGUAGE , "English" ) AND LIMIT-TO ( DOCTYPE , "ar" )

**4. Web of Science (Core Collection)** *Parameters applied: Topic (TS) field.*

TS=(("HPV vaccin*" OR "cervical cancer screening" OR "pap smear") AND ("chatbot*" OR "conversational agent*" OR "conversational AI" OR "serious game*" OR "interactive app*" OR "digital assistant*")) *Refined by: Document Types: (Article) AND Languages: (English) AND Publication Years: (2016-2026)*

**5. Cochrane Central Register of Controlled Trials (CENTRAL)** *Parameters applied: MeSH descriptors exploded and combined with Title/Abstract/Keyword text.*

#1 MeSH descriptor: [Papillomavirus Vaccines] explode all trees #2 MeSH descriptor: [Early Detection of Cancer] explode all trees #3 ("HPV vaccin*" OR "cervical cancer screening"):ti,ab,kw #4 #1 OR #2 OR #3 #5 MeSH descriptor: [Artificial Intelligence] explode all trees #6 ("chatbot*" OR "conversational agent*" OR "serious game*" OR "interactive digital"):ti,ab,kw #7 #5 OR #6 #8 #4 AND #7 (Limit to Publication Year 2016-2026)

**6. SpringerLink** *Parameters applied: Full-text keyword proximity search due to platform-specific Boolean limitations.*

("HPV vaccine" OR "cervical cancer screening") AND ("chatbot" OR "conversational AI" OR "serious game" OR "interactive app") *Filters Applied: Discipline (Medicine & Public Health), Content Type (Article), Language (English), Year (2016-2026).*
